# Predictive Methods for Thrombus Formation in the Treatment of Aortic Dissection and Cerebral Aneurysms: A Comprehensive Review

**DOI:** 10.3390/bioengineering11090871

**Published:** 2024-08-28

**Authors:** Kenji Komiya, Shuta Imada, Yoshihiro Ujihara, Shukei Sugita, Masanori Nakamura

**Affiliations:** Department of Electrical and Mechanical Engineering, Graduate School of Engineering, Nagoya Institute of Technology, Nagoya 466-8555, Japan; s.imada.079@stn.nitech.ac.jp (S.I.); ujihara.yoshihiro@nitech.ac.jp (Y.U.); sugita.shukei@nitech.ac.jp (S.S.); nakamura.masanori@nitech.ac.jp (M.N.)

**Keywords:** thrombogenesis, aortic dissection, cerebral aneurysm, mathematical model, simulation, prediction

## Abstract

Thrombus formation plays a crucial role in the clinical treatment of certain diseases. In conditions such as aortic dissection and cerebral aneurysm, complete thrombus occlusion in the affected region is desired to reduce blood flow into the false lumen or aneurysm sac, leading to a decrease in the tension exerted on the vascular wall and making it less likely to rupture. However, desired thrombosis sometimes fails to occur. Predicting thrombus formation can provide valuable information in such cases. This article offers a comprehensive review of conventional methods for predicting thrombus formation. In reviews conducted from the year 2000 to the present, the number of published related papers every five years has increased more than tenfold. We also found that the predictive methods can be classified into two categories: those based on the hemodynamic evaluation parameters and those based on hemodynamic and mathematical models that simulate the transport and reaction of blood components. Through our discussions, we identified several challenges that need to be resolved, including predictions based on patient-specific condition, model validation, multi-scale problems, the mechanisms of thrombus formation, and ensuring cost effectiveness. This review aims to guide researchers interested in exploring thrombus formation prediction within clinical treatments.

## 1. Introduction

Thrombus is a coagulation of blood. Normal thrombogenesis happens to repair damaged blood vessel walls: components in the blood coagulate and adhere to the site of vessel wall damage, forming a solid clot to prevent further blood loss and additional damage to the vessel wall while facilitating its repair.

Some cardiovascular diseases involve thrombogenesis in their clinical treatment. The challenge with these treatments lies in the difficulty of controlling thrombus formation. Many treatments aim for complete thrombogenesis of the targeted site. However, some cases end with partial thrombus formation even after months or years of treatment. In such cases, the dissected lumen and the aneurysm remain at risk of rupture, leading to increased patient burden and the cost of potential additional surgeries [1].

Thrombogenic prediction could be a powerful tool in determining treatment strategies for diseases that involve thrombus formation. High-accuracy thrombogenesis prediction allows for the consideration of treatment policies based on reliable evidence and gives a chance to change the uncertain treatment policies based on the experience and intuition of physicians. Tremendous efforts have been poured into predicting thrombogenesis during disease treatment for this purpose. However, despite such efforts, the clinical application of thrombogenesis prediction has not yet been fully realized [2].

This review aims to clarify the issues that need to be resolved for the clinical application of thrombogenic prediction. To this end, it first clarifies today’s understanding of the mechanisms of thrombogenesis related to treatment. Then, it investigates diseases that involve thrombogenesis as part of their treatment and specifies the requirements for thrombogenesis prediction technology. Finally, through a comprehensive survey of current thrombogenesis prediction technology, we describe unresolved issues within the current state of the technology and discuss the direction of future research.

## 2. The Mechanism of Thrombosis

Thrombus formation involves three factors: changes in hemodynamics, changes in the blood vessel wall, and changes in blood components, as represented by Virchow’s triad [3]. Changes in blood components, encompassing alterations in viscosity and coagulation factors, are pivotal in clot formation. Real-world examples abound, such as in polycythemia vera, where an excess of red blood cells increases viscosity, heightening the risk of thrombotic events [4]. Changes in the blood vessel wall include endothelial cell injury and arteriosclerosis. Endothelial dysfunction disrupts the delicate balance between anticoagulant and procoagulant properties, fostering clot formation. Atherosclerosis, often associated with endothelial disruption and plaque rupture, exemplifies this phenomenon, as seen in coronary or cerebral arteries, where it precipitates myocardial infarction or stroke, respectively [5]. Disturbances in blood flow patterns are represented by stagnation, recirculation, and turbulence. Stagnant blood flow in dilated veins or heart chambers increases the risk of clot formation by reducing shear forces that prevent platelet adhesion. In cases of high shear rates, typical in narrowed vessels or turbulent flow conditions, platelets are activated, and thrombus formation is promoted. Real-world instances include deep vein thrombosis, where sluggish blood flow in the lower extremities combined with endothelial injury or hypercoagulable states leads to clot formation [6].

Thrombi formed by these factors are classified into two types: arterial thrombi and venous thrombi. The difference between these two types of thrombi is derived from their formation mechanisms, with changes in the blood vessel wall being most crucial for arterial thrombi, and changes in blood flow being most crucial for venous thrombi. Some cardiovascular treatments often promote blood stagnation with the hope of thrombus formation. Such stagnation-induced thrombosis is similar to the characteristics of venous thrombi. 

**Figure 1:**
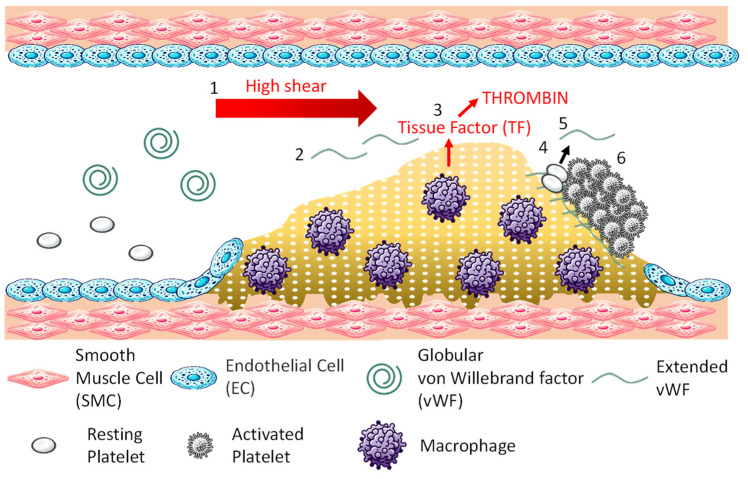
Mechanism of arterial thrombosis.

An overview of arterial thrombosis is shown in Figure 1. Arterial thrombosis is often caused by the rupture of atherosclerotic plaques. The development and growth generally proceed in the following six stages: 1. High shear rates occur at the site of vascular narrowing, usually caused by the atherosclerotic plaque; 2. von Willebrand factor (vWF) unfolds in a region of high shear rates, exposing binding sites for collagen and platelets [7]; 3. Plaque rupture exposes the underlying extracellular matrix collagen (additionally, macrophages within the plaque express tissue factor, initiating thrombin formation and the coagulation response [8]); 4. Exposed collagen binds vWF, and platelets adhere to vWF [9]; 5. When the adhered platelets become activated, they release vWF from their dense granules [10]; 6. The released vWF binds to platelets on the plaque, and further, platelets release vWF, repeating this process to facilitate thrombus growth. Arterial thrombi are referred to as white thrombi since they contain a high concentration of colorless platelets.

**Figure 2:**
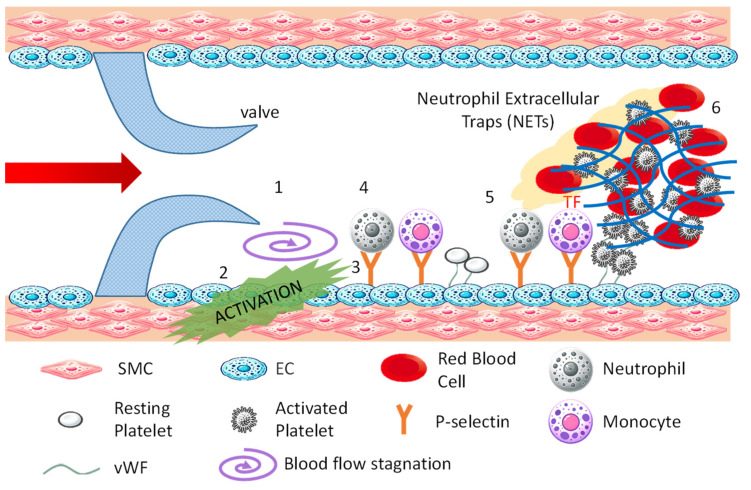
Mechanism of venous thrombosis.

Figure 2 is a schematic drawing of the formation process of venous thrombi. The process unfolds as follows: 1. Blood flow stagnates (purple swirl arrow) in the area behind the venous valve (valve pocket); 2. The stagnation of flow activates endothelial cells [11]; 3. Activated endothelial cells express P-selectin, a membrane protein stored in Weibel–Palade bodies within the cell, on their surface, allowing vWF to anchor on the cell surface [12]; 4. Neutrophils, monocytes, and platelets in the blood bind to adhesion factors on the surface of activated endothelial cells [13]. Neutrophils and monocytes bind to P-selectin, and platelets bind to vWF; 5. After binding to P-selectin, neutrophils release neutrophil extracellular traps (NETs), which are composed of chromatin DNA, histones, and antimicrobial substances, promoting thrombus formation by entangling platelets and red blood cells [14]. After binding to P-selectin, monocytes express tissue factor and initiate the extrinsic coagulation cascade. Platelets bound to vWF are activated by thrombin, releasing adenosine diphosphate and polyphosphates from granules, which further activate surrounding platelets. They also release vWF and fibrinogen to promote platelet aggregation; 6. The extrinsic and intrinsic coagulation reactions initiated by monocytes and neutrophils lead to the formation of a fibrin network to capture blood components, especially red blood cells, stabilizing the thrombus. Venous thrombi are known as red thrombi because they are rich in red blood cells. 

Unlike arterial and venous thrombi, thrombi can also form due to artificial interventions involving foreign biomaterials. The trigger for this type of thrombus formation is the adsorption of plasma proteins [15], particularly fibrinogen, onto the bio-material’s surface, which initiates a multi-step biological response [16] seen in venous thrombi, especially in steps 4–6, where platelet adhesion, activation, and thrombus growth occur. 

It should be noted that thrombus formation is generally undesirable due to its association with severe medical conditions. A thrombus in an artery can restrict or completely block blood flow, leading to critical issues such as heart attacks, strokes, or damage to other organs, depending on the location [17]. Venous thromboembolism, where thrombi in veins dislodge and travel to the lungs, can cause life-threatening pulmonary embolism [18]. Additionally, deep vein thrombosis can result in post-thrombotic syndrome, characterized by chronic pain, swelling, and skin changes.

## 3. Treatments of Cardiovascular Diseases Involving Thrombosis

In some cardiovascular diseases, such as aortic dissections and cerebral aneurysms, medical doctors perform treatments that intentionally induce thrombus formation to prevent further progression of diseases and better prognosis. This section outlines aortic dissection and cerebral aneurysms and their treatment methods. It also examines the current challenges in treating these diseases and clarifies methods for improving treatment.

### 3.1. Treatment of Aortic Dissection and Thrombosis Formation

Aortic dissection is an emergency cardiovascular condition where the medial layer of the aortic wall splits into two layers. The original vascular lumen is called the true lumen, and the lumen formed by the dissection is called the false lumen. Dissections can progress extensively along the aorta and its branches, causing rupture or branch perfusion problems. The mortality rate within 24 h of onset is 50%, making it a highly dangerous condition [19].

Thoracic endovascular aortic repair (TEVAR) is the technique of placing a stent graft in the aorta for the treatment of aortic dissection [20]. TEVAR was initially used to provide treatment to patients who were not considered to be surgical candidates, but it is now preferentially chosen for the treatment due to less invasiveness and improved outcomes compared with open-chest surgery. TEVAR provides a mechanical support to the aortic wall and prevents direct imposition of blood pressure to the aortic wall. In addition, TEVAR seals the entry of the dissection (passage from the true lumen to the false lumen) with a stent graft. This obstructs blood flow from the true lumen to the false lumen, promoting thrombogenesis by causing blood stagnation. Complete thrombogenesis of the false lumen leads to its reduction [21], while partial thrombogenesis can cause the false lumen to expand, posing a risk of rupture [22].

### 3.2. Treatment of Cerebral Aneurysms and Thrombosis Formation

A cerebral aneurysm is a condition where a part of a cerebral artery bulges out. Approximately 6% of the total population has cerebral aneurysms [23,24]. Rupture of a cerebral aneurysm causes in a potentially life-threatening subarachnoid hemorrhage, which results in a 20% mortality rate [25]. 

There are two main treatment methods for unruptured cerebral aneurysms. One is open surgery, which involves removing a portion of the skull and directly closing off the aneurysm with a clip. Open surgery is highly reliable but imposes a significant physical burden on the patient. The other is endovascular treatment, which involves placing devices within the blood vessels through a catheter. Endovascular treatment is less invasive compared to open surgery and has been increasingly chosen in clinical settings [26]. 

The goal of endovascular treatments is to induce thrombus within the aneurysm. Representative methods of endovascular treatments include the placement of coil embolization and a flow diverter (FD). The coil treatment for cerebral aneurysms involves placing tiny coils made of soft platinum wire into the aneurysm through a catheter. Once deployed, these coils fill the aneurysm, obstructing blood flow into it and promoting thrombogenesis within the aneurysm sac. An FD is a device made of metal wires woven into a mesh, which is installed in the parent vessel to cover the aneurysm neck. FD treatment also obstructs blood flow from the parent vessel to the aneurysm, causing blood stagnation and thus inducing thrombogenesis. FD treatment is known to be more effective for treating large aneurysms compared to coil embolization. The thrombus caused by FD and coil treatment inhibits blood flow within the aneurysm and protects the aneurysm wall, preventing its rupture.

### 3.3. Challenges and Requirements for Resolution

A common challenge in the treatment of aortic dissection and cerebral aneurysms is the failure to form the targeted thrombus. Even if the treatment is technically successful, some cases end up with partial or no thrombus formation [27,28,29,30]. In these cases, since the flow of blood into the false lumen and aneurysm is maintained, both remain at continued risk of rupture. It is not known which treatment methods will achieve the targeted thrombosis, necessitating reliance on the experience and intuition of physicians for the application and selection of treatment. This increases the risk and burden on patients and the costs associated with potential additional surgeries. 

To address this challenge, there is a need for clinically applicable technologies to predict thrombus formation. If future thrombus formation can be identified before treatment, it will be possible to avoid the risk of incomplete thrombus formation and to consider therapeutic methods that more effectively induce desired thrombus formation.

## 4. Efforts on Thrombosis Prediction in Aortic Dissection and Cerebral Aneurysm

**Figure 3:**
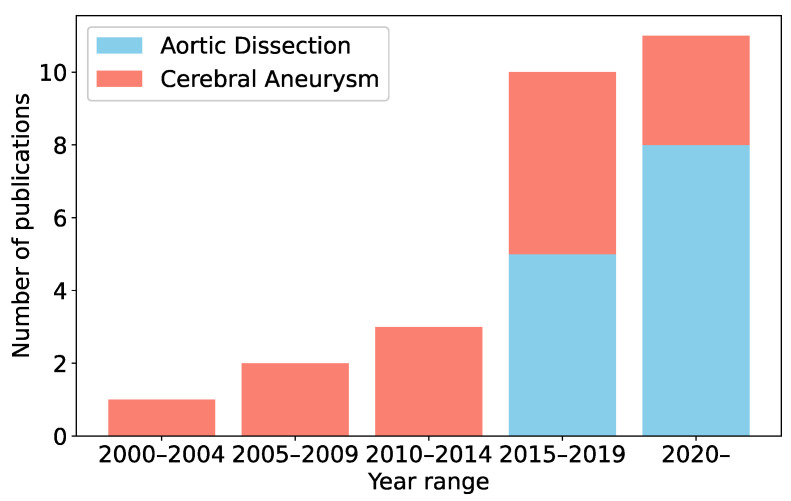
Publication counts related to thrombus formation simulation every five years from 2000 to the present.

This section investigates the literature concerning the prediction of thrombus formation in aortic dissection and cerebral aneurysms. Research was conducted using the Google Scholar search engine. We used a combination of keywords, including “aortic dissection”, “cerebral aneurysm”, “thrombus formation”, “thrombogenesis”, “clot development”, “intracranial aneurysm”, “simulation”, prediction”, “forecast”, and “mathematical model”. The literature relevant to the target diseases and the prediction of thrombus formation was selected based on content alignment. In this review, we focused on prediction methods that use simulations incorporating the mechanisms of thrombus formation. Consequently, we excluded papers that statistically analyzed the relationship between thrombus formation and factors not directly related to the formation process, such as patients’ personal data, including weight and age, and vascular geometry data, such as diameter and curvature. In Figure 3, the publication frequency of the selected literature is illustrated over five-year intervals. The field began to gain attention with a seminal paper on cerebral aneurysms published in 2002 [31]. Subsequently, the volume of related publications has increased every five years. In contrast, the first significant studies on aortic dissection appeared later, with the earliest relevant publication emerging in 2016 [32]. Prior to this, some studies highlighted the prognostic relationship between thrombus formation and aortic dissection outcomes [2,33], enhancing the perceived importance of thrombus formation in managing aortic dissection.

Further analysis included the nationalities of the first authors’ affiliated organizations. The United Kingdom emerged as the leading contributor, with over 25% of the publications originating there, particularly from a prominent team at Imperial College London. This underscores their pivotal role in advancing this research area. Additionally, significant contributions have come from institutions in China, the USA, Spain, Malaysia, Switzerland, Japan, Belgium, and Austria. The global spread of research, spearheaded by institutions such as Imperial College London, underscores the increasing recognition of the need for advanced thrombus formation prediction.

## 5. Thrombosis Prediction Methods

Some studies predict thrombotic regions based on hemodynamics [1,30,31,34,35,36,37,38,39,40,41,42,43]. Many studies have focused on blood stagnation, which is a hallmark of venous thrombosis, evaluating it through the flowrate into the aneurysm sac and false lumen [1,34,35,39]; the velocity magnitude of the blood flow [1,42]; the kinetic energy [34,35]; or the residence time (RT)—the time particles remain within the fluid domain [31,36,38,41,43]. Wall shear stress (WSS) has also garnered attention due to multiple studies indicating its association with the thrombogenesis and prognosis of diseases [30,35,36,37,41,43]. There is a study that focuses on the oscillatory shear index (OSI), which is calculated based on the temporal variation of WSS over one cardiac cycle [38]. Furthermore, vortex structures are examined due to their importance in platelet transport [37], and shear rates are analyzed for their role in platelet adhesion, both to each other and to the vascular wall [40]. Most studies in this area set thresholds by comparing the distribution of these indicators within vessels to actual thrombotic regions in patients, thereby defining boundaries between blood and thrombotic areas [30,31,36,37,38,40,41,42,43]. Some studies perform binary predictions of thrombus formation based on the volumetric mean of indicators based on hemodynamics [34,35,39]. These prediction methods are handy once good indicators of thrombogenesis are identified. However, they do not consider the biochemical reactions that govern the sequential progression of clot formation. As a result, the resulting information is limited to a single near-future point in time.

Simulations based on the biochemical cascade of thrombogenesis can provide a time-dependent thrombus formation process. These simulations define the reaction and transport of blood components as mathematical models [32,44,45,46,47,48,49,50,51,52,53,54]. Menichini et al. [51] pioneered the application of these simulations to thrombus formation in aortic dissection. Their proposed model includes variables corresponding to platelet and coagulant concentrations. Platelets transition through resting, activated, and bound states in a one-way process. The source term in the advection–diffusion equation controls the chemical changes in blood components. Increased concentrations of bound platelets indicate the formation of a thrombus, which increases the viscosity and resistance of blood flow. This mathematical model was validated using both idealized and patient-specific aortic dissection conditions [32,48,50,51]. Following the proposal of this mathematical model–based simulation, various studies were conducted in this area. Wang et al. [44] proposed a mathematical model that can simulate thrombus breakdown in addition to thrombogenesis based on shear rate. The simulation results indicate that the proposed model can predict outcomes more consistently with experimental results compared to conventional models. Chong et al. [46] conducted a fluid–structure interaction (FSI) simulation study to model the interaction between hemodynamics and the deformation of the flaps in aortic dissection. FSI simulation is a sophisticated computational method that simultaneously considers the dynamic interplay between fluid flow (such as blood) and the structural response of tissues (such as vessel walls). By interactively solving the governing equations of both fluid and structure, FSI provides a more accurate representation of physiological conditions. The FSI simulation results indicated a greater thrombus volume when compared to the traditional rigid wall assumption, highlighting the importance of accounting for structural deformation in such models. There are differences in the number of blood component species considered in each mathematical model. Menichini et al. [51] considered three species of platelets and one species of coagulant, representing a coarse-grained version of the known components and reactions. As previous studies have shown, more chemical factors are involved in thrombus formation [55,56]. To describe the coagulation process more precisely, some studies consider additional components. For example, Wang et al. [47] and Ou et al. [53] included more than twenty components in their models. Conversely, Jafarinia et al. [45] demonstrated that models with fewer blood components can perform similarly to those with more components, suggesting that more detailed models do not always lead to improved simulation results. Ngwenya et al. [54] applied a model that focused solely on the transport and reaction of fibrin within two-dimensional cerebral aneurysm shapes. When compared with in vitro experimental results, this model demonstrated a similar increase in the rate of thrombus occlusion in cerebral aneurysms. These methods would be effective for long-term predictions of thrombogenesis, since they considered hemodynamic changes brought about by thrombus formations. Challenges lie in determining the values of parameters such as diffusion coefficients and reaction rates. 

Through this review, we identified two main categories of thrombus formation prediction methods. The first category is based on hemodynamic evaluation parameters, while the second relies on hemodynamics and mathematical models that simulate the transport and reaction of blood components. The key difference between these approaches lies in their handling of time-dependent interactions between hemodynamics and blood components, especially platelets. Methods using the mathematical models can predict time-dependent changes in thrombus geometry by simulating interactions between hemodynamics and biochemical processes, whereas the hemodynamic parameter–based method predicts thrombus formation at an unspecified future point. The number of required parameters for each method also differs. Predictive methods based solely on hemodynamics parameters typically require only the threshold values that determine whether or not thrombus formation takes place. In contrast, the methods that takes account of the transport and reactions of blood components require more parameters to be set, such as diffusion coefficients and reaction coefficients for each blood component, many of which are not directly measurable.

**Figure 4:**
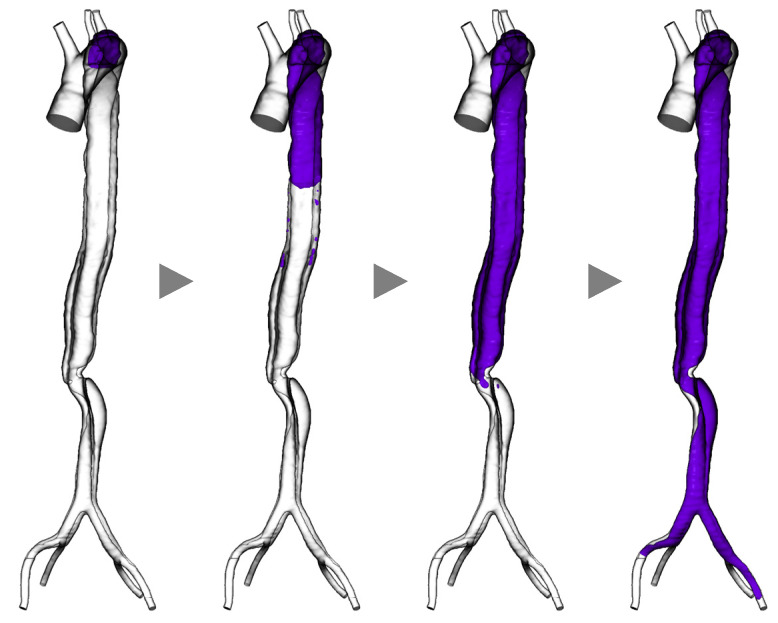
Simulation results of thrombogenesis in a case of aortic dissection (purple indicates the thrombosed region, grey arrow heads indicate the progress of time within simulation).

We applied a method inspired by Menichini et al.’s mathematical model [51] to a clinical case of Stanford type B aortic dissection in which the false lumen thrombosed during follow-up. Stanford type dissection is a classification of aortic dissection based on the location of the tear. Type A involves a tear in the ascending aorta, while Type B is characterized by tears in the aortic arch and descending aorta, with no involvement of the ascending aorta. In cases of Stanford type B dissection, conservative treatment is often pursued [57]. Figure 4 presents a sequence of representative images where we applied the model to a clinical case of aortic dissection. The thrombus (purple region) initially formed in the false lumen near the entry located at the distal end of the aortic arch. With the advancement of the simulations, the thrombus developed downward up to the common iliac arteries and eventually occluded the false lumen, while the arch and true lumen remained patent. Such a thrombus formation process is congruent with what is clinically observed. Providing time-series information is a crucial aspect of these methods.

## 6. Unresolved Issues and Future Directions

We propose five key challenges to enhancing the availability of thrombosis prediction systems in clinical practice: patient-specific condition setting, system validation, time-scale modeling, understanding the underlying mechanisms, and ensuring cost effectiveness. 

The first challenge involves adapting the prediction model to patient-specific conditions. Simulations using the prediction model require the setting of multiple parameters. Specifically, in hemodynamic-chemical-reaction models, examples include the threshold of the activation function governing platelet changes, the coefficient determining the rate of chemical reactions, and the concentration of platelets in the inflowing blood. These parameters cannot be directly measured from patients at present. Although assumed values are used in practice, past sensitivity analyses have demonstrated that parameters significantly affect simulation outcomes [51]. This underscores the necessity of setting these parameters based on the patient’s condition. One approach to accomplish this involves identifying the relationship between the clinical information of a patient and the parameters. Previous research indicated that a patient’s smoking history affected the levels of fibrinogen and vWF in the blood, both of which are involved in thrombus formation. In investigations conducted by Liu [58] and colleagues, the fibrinogen concentration in non-smokers was found to be 305.18 mg/dL, compared to 325.55 mg/dL in smokers. Similarly, vWF levels were 100.56% in non-smokers versus 108.39% in smokers.

Based on an analysis of past research, we identified two problems related to the validation of the model. In general, the number of cases compared with simulations tends to be limited to approximately 1–3 cases. This limitation leads to a lack of evidence that the same methodology is effective across different patients. Moreover, the postoperative time point for comparing prediction results is restricted to a single point. This brings difficulties to validating temporal changes in thrombus formation predicted by the model. To address this issue, it is essential to construct a more comprehensive dataset of a larger number of patients over time. Ideally, the data would cover a long-term period of more than three years to adequately represent the timescale at which thrombus formation progresses.

There are also issues concerning the timescale of models and simulations. Thrombus formation is a phenomenon that occurs over several months to years. In contrast, the blood components and hemodynamics that influence the mechanism of thrombus formation change within a much shorter timeframe, often in seconds or less. Such overwhelming differences in timescale pose significant challenges to the mathematical modeling of thrombus formation. To resolve this issue, a deeper insight into how short-term variations in thrombogenic factors affect long-term thrombus formation is required, along with their accurate mathematical modeling.

In addition to the challenges mentioned above, elucidation of the mechanisms of thrombus formation for each disease and treatment method is a crucial element. The mechanisms of thrombus formation typically involve hemostatic and venous thrombi, which have been deeply investigated in physiology and pathology. The process by which thrombi form during the actual treatment of aortic dissection and cerebral aneurysms is not necessarily the same and may include undiscovered pathways. In vivo and in vitro experiments will help find thrombogenic mechanisms associated with each disease and treatment.

Predictions using mathematical models or CFD require significant computational resources. For instance, the example presented in Section 5 demanded a high-performance computer and over a day’s processing time. However, in clinical practice, acquiring and maintaining such resources can be challenging due to budget constraints. Cloud computing [59] offers a solution by allowing users to share computational resources over a network, eliminating the need for individual hardware ownership. This approach makes advanced computing more accessible and cost effective, enabling broader adoption in clinical settings.

Future research should aim to develop more accurate and patient-specific thrombus formation prediction models, validate them based on clinical data, and aim for the clinical application of these prediction technologies. This is expected to increase the success rate of treatments and improve patient outcomes.

## 7. Conclusions

This review focuses on diseases in which thrombosis occurs during the treatment process, especially aortic dissection and cerebral aneurysms, and investigated thrombus formation prediction methods. Our review identifies two primary methods for predicting thrombus formation: one based on analysis of hemodynamics evaluation parameters and the other based on hemodynamics and mathematical models that simulate the transport and reaction of blood components. However, several challenges must be addressed to ensure availability for clinical application. We categorize these challenges into patient-specific conditions, model validation, multiscale modeling, understanding the mechanisms of thrombogenesis, and ensuring cost effectiveness. By overcoming these challenges, we can unlock the full potential of thrombus formation prediction technology, transforming it into a vital tool that significantly enhances clinical outcomes and patient care.

## Data Availability

Data are contained within the article.
